# Laparoscopic resection of a metastatic myxoid liposarcoma in the mesentery of the small intestine: a case report

**DOI:** 10.1186/s40792-023-01715-7

**Published:** 2023-07-21

**Authors:** Fumika Kamehama, Tatsuya Kinjo, Yoshihiro Miyagi, Tomonori Furugen, Takao Teruya, Tomoko Tamaki, Naoki Wada, Mitsuhisa Takatsuki

**Affiliations:** 1 Department of Digestive and General Surgery, Graduate School of Medicine, University of the Ryukyus, 207 Uehara, Nishihara, Okinawa 903-0215 Japan; 2 Department of Thoracic and Cardiovascular Surgery, University Hospital of The Ryukyus, 207 Uehara, Nishihara, Okinawa 903-0215 Japan; 3 Department of Pathology and Oncology, Graduate School of Medicine, University of the Ryukyus, 207 Uehara, Nishihara, Okinawa 903-0215 Japan

**Keywords:** Myxoid liposarcoma, Distant metastases, Small intestinal mesentery, Laparoscopic resection

## Abstract

**Background:**

Myxoid liposarcoma (MLS), with its risk factors, tends to spread to the lungs and extraperitoneally, with intraperitoneal metastases occurring rarely. We present an unusual case of a myxoid liposarcoma that metastasized to the abdominal organs.

**Case presentation:**

A 60-year-old female patient was referred to our hospital for the evaluation of a right upper limb tumor that had been growing for 7 years. The patient refused surgery, and during follow-up, tumor hemorrhage resulted in hemorrhagic shock. The patient’s right upper limb was immediately amputated. MLS was diagnosed histopathologically. Subsequently, the patient underwent adjuvant chemotherapy. Computed tomography (CT) revealed a right buttock mass, a pelvic mass, and left cardiophrenic angle lymph nodes 3 years after the initial surgery. Contrast-enhanced abdominal CT revealed a relatively low-density, lobulated pelvic tumor. Contrast-enhanced pelvic magnetic resonance imaging (MRI) revealed a low-intensity, lobulated mass on T1-weighted images and a high-intensity mass on T2-weighted images. The pelvic mass showed no significant fluorodeoxyglucose (FDG) uptake on positron emission tomography (PET)-CT. On clinical examination, gynecological malignancies were ruled out as the origin of the pelvic lesions. After resection of the right buttock mass, pelvic mass, and left cardiophrenic angle lymph nodes, the patient underwent laparoscopic surgery for a preoperative diagnosis of small intestinal mesenteric metastasis of MLS. A tumor was found in the mesentery of the small intestine and removed with a margin of 5 cm on both the proximal and distal sides. The specimen measured 10 × 8 × 5 cm and contained a multifocal mass. The tumor was found in the mesentery of the small intestine, with no mucosal or submucosal invasion. The patient was diagnosed with MLS with small mesenteric intestinal metastases. On postoperative day 8, the patient was discharged after an uneventful postoperative course. Twelve months after the surgery, there was no evidence of local or distant recurrence.

**Conclusions:**

Small intestinal mesenteric metastases of MLSs are rare. Moreover, there are few reports on laparoscopic resection. In this case, the laparoscopic approach was useful in detecting the tumor location and determining the range of resection.

## Background

Liposarcoma is the most common malignant soft tissue tumor. Myxoid liposarcoma (MLS), a subtype of liposarcoma [1], generally manifests in the lower extremities [2]. Metastatic tumors, which typically develop in the lungs, paravertebral regions, retroperitoneum, contralateral extremities, and extraperitoneal sites such as the axilla and bones, affect approximately one-third of patients with MLS [1, 3]. However, reports of intraperitoneal metastases are rare [2]. Complete resection has been shown to considerably improve the prognosis of patients with metastases, although the optimal treatment strategy for metastatic MLS remains unknown [1, 3]. Small intestinal mesenteric metastases from MLS are rare. Moreover, few laparoscopic resections have been reported [4]. We present a rare case of laparoscopic excision of a small intestinal mesenteric metastasis of MLS, along with a literature review.

## Case presentation

**Fig. 1:**
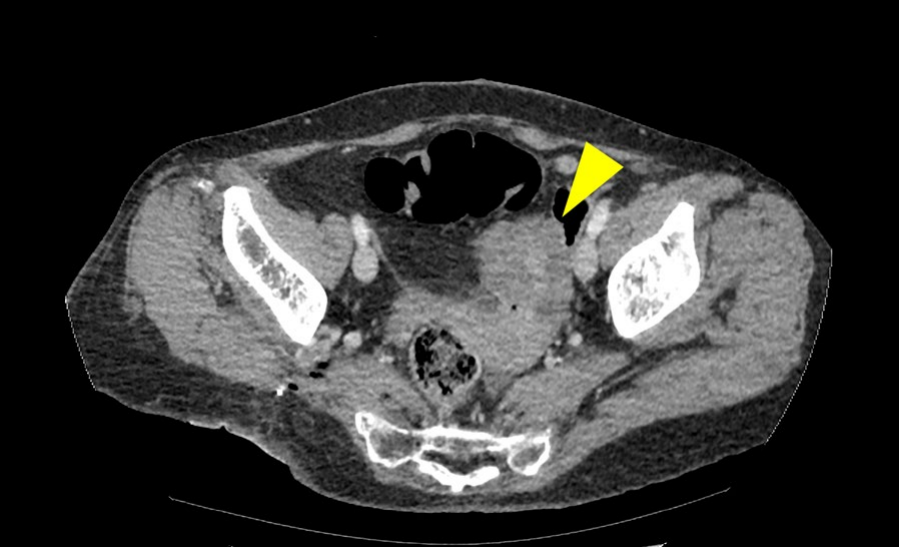
A contrast-enhanced CT scan showing a low-density lobulated tumor in the pelvis adjacent to the small intestine

**Fig. 2:**
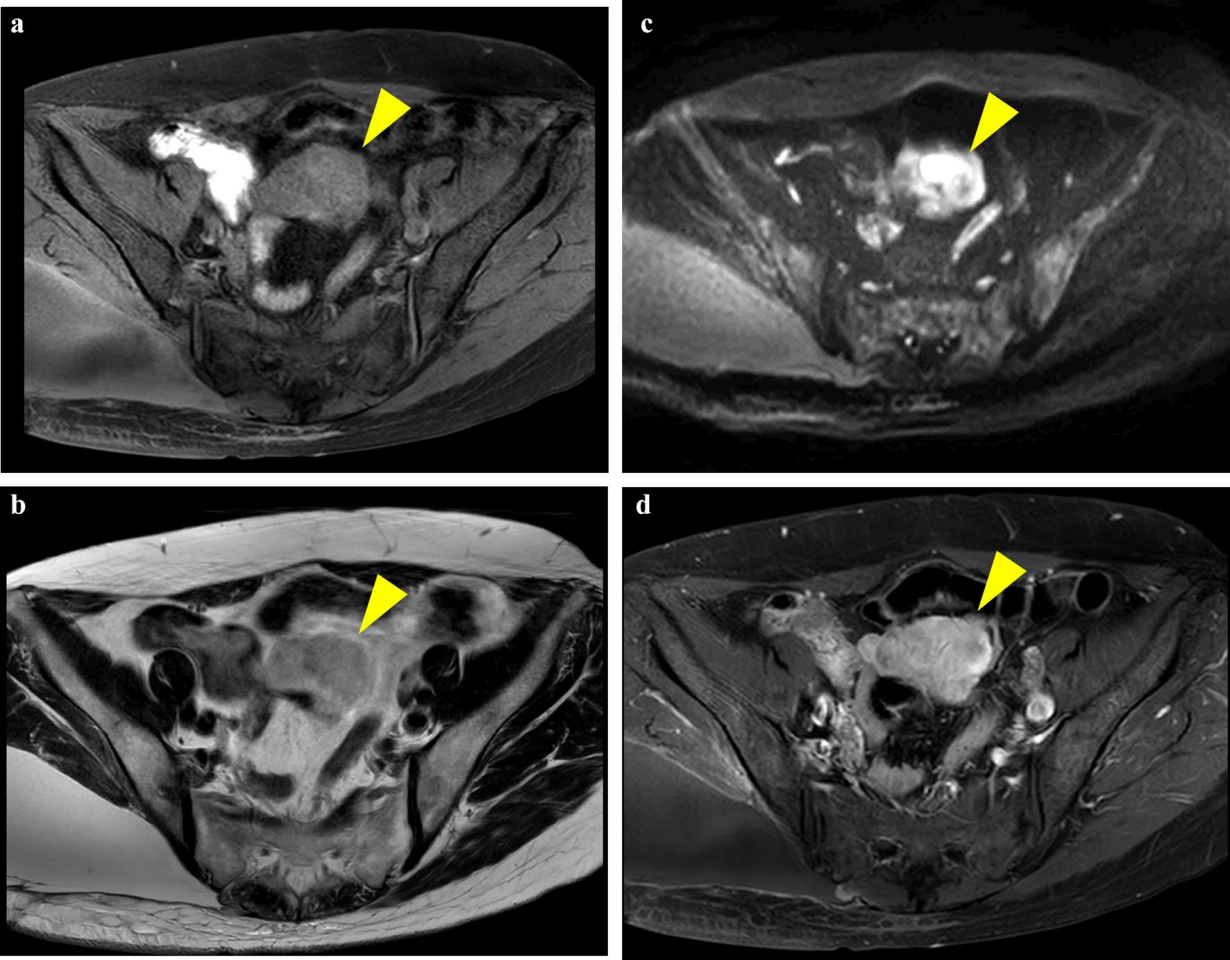
Magnetic resonance imaging showing a lobulated mass with **a** low intensity on T1-weighted and **b** high intensity on T2-weighted images. **c** Magnetic resonance imaging diffusion-weighted images showing diffusion restriction of the mass. **d** Contrast-enhanced magnetic resonance imaging showing contrast enhancement of the mass

A 60-year-old female patient presented to our hospital for evaluation of a right upper extremity tumor that had been present for 7 years. The patient declined surgery and developed hemorrhagic shock due to tumor hemorrhage during follow-up. An emergency right upper extremity amputation was performed. Histopathological examination revealed MLS. Subsequently, the patient underwent adjuvant chemotherapy, and two cycles of doxorubicin and ifosfamide were administered for 21 days. Computed tomography (CT) performed 3 years after the initial surgery revealed masses in the left cardiophrenic angle, right buttock, and pelvis. On clinical examination, gynecological malignancies were ruled out as the origin of the pelvic lesions. Abdominal contrast-enhanced CT revealed a relatively low-density lobulated pelvic tumor anterior to the uterus. The small intestine was located adjacent to the mass (Fig. 1). Contrast-enhanced magnetic resonance imaging (MRI) of the pelvis revealed a low-intensity, lobulated mass on T1-weighted images and a high-intensity mass on T2-weighted images. Diffusion-weighted images showed diffusion restriction, and contrast studies showed contrast enhancement of the mass (Fig. 2a–d).

**Fig. 3:**
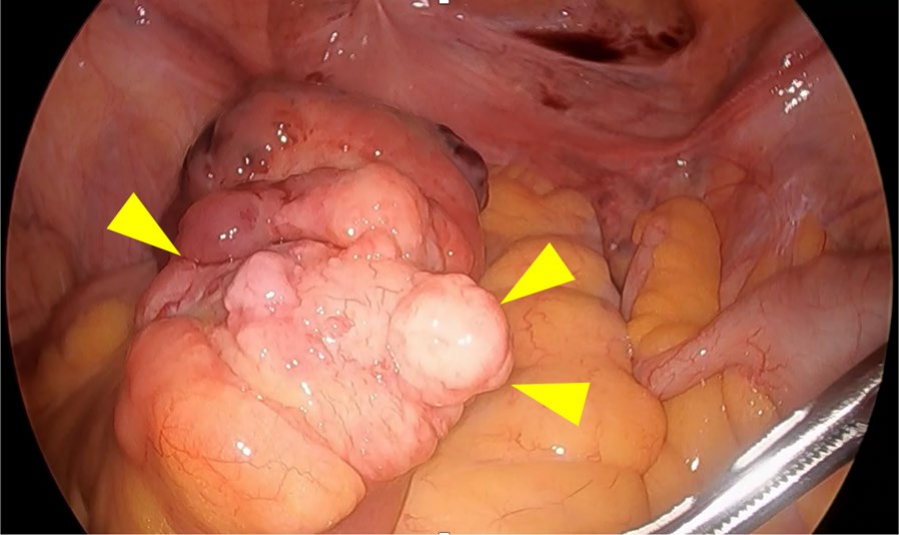
A tumor located in the mesentery of the small intestine 60 cm proximal to the Bauhin valve

Positron emission tomography (PET) showed no significant fluorodeoxyglucose (FDG) accumulation in the pelvic mass. First, the cardiophrenic angle tumor, found in the epipericardial fat tissue with slight adhesion to the pericardium, was resected completely by a thoracic surgeon. Secondly, the right buttock tumor, contained in the gluteus maximus muscle, was resected with 2 cm margins by an orthopedic surgeon. Finally, the preoperative diagnosis was small intestinal mesenteric metastasis of MLS, and the patient underwent metachronous laparoscopic surgery under general anesthesia in the lithotomy position. Initially, diagnostic laparoscopy was performed to evaluate the presence of superficial liver and peritoneal metastases. The tumor was located in the mesentery of the small intestine 60 cm proximal to the Bauhin valve (Fig. 3). The tumor was extracorporeally resected with 5 cm margins on both the proximal and distal sides.

**Fig. 4:**
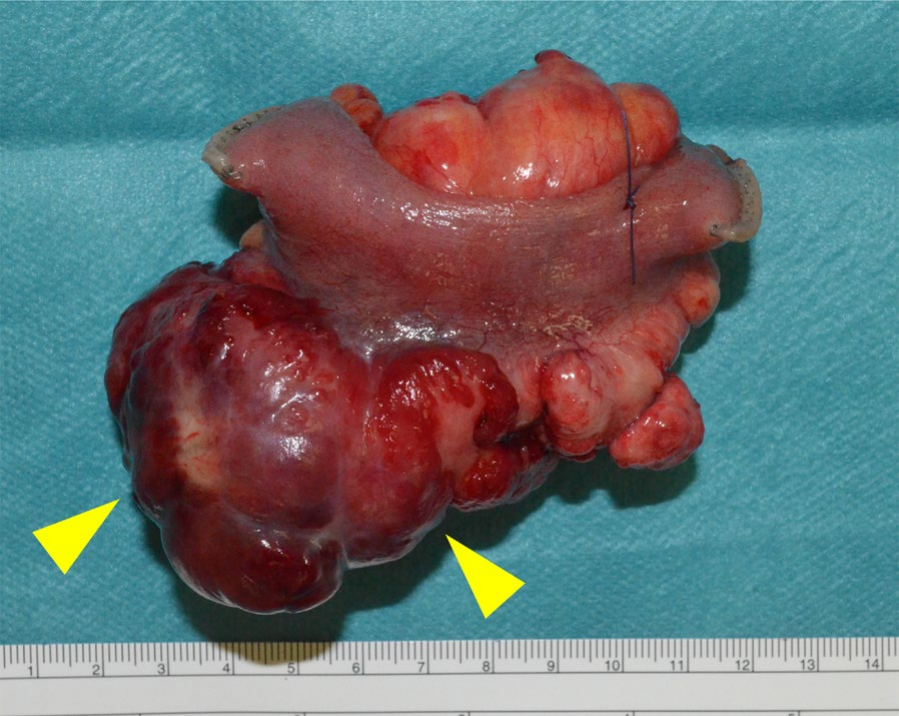
The resected 10 × 8 × 5 cm multifocal mass containing specimen without invasion into the mucosal surface of the small intestine

**Fig. 5:**
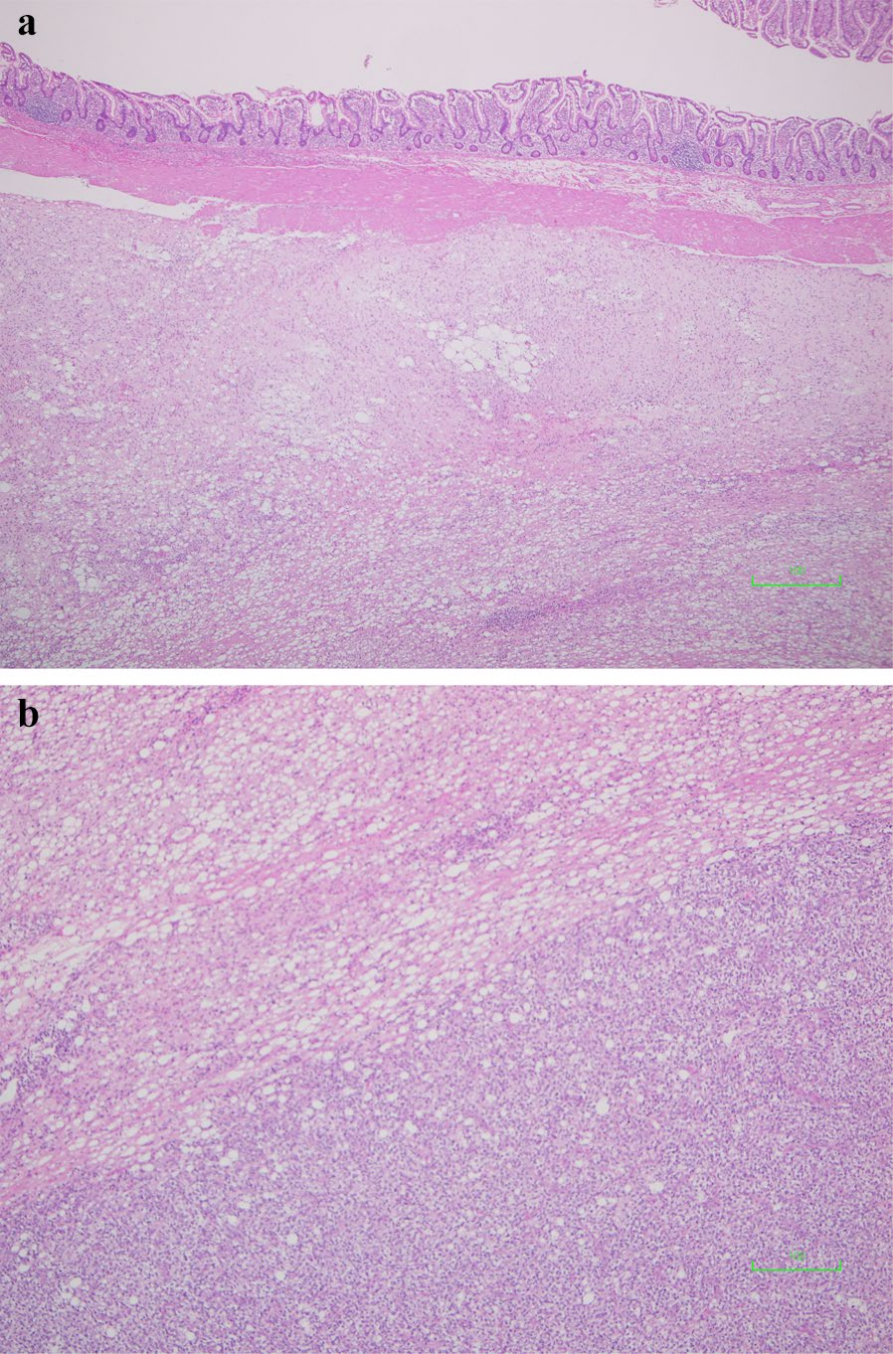
**a**, **b** Histopathological examination of the mass showing mostly atypical cells with clear nucleoli and increased cell density

**Fig. 6:**
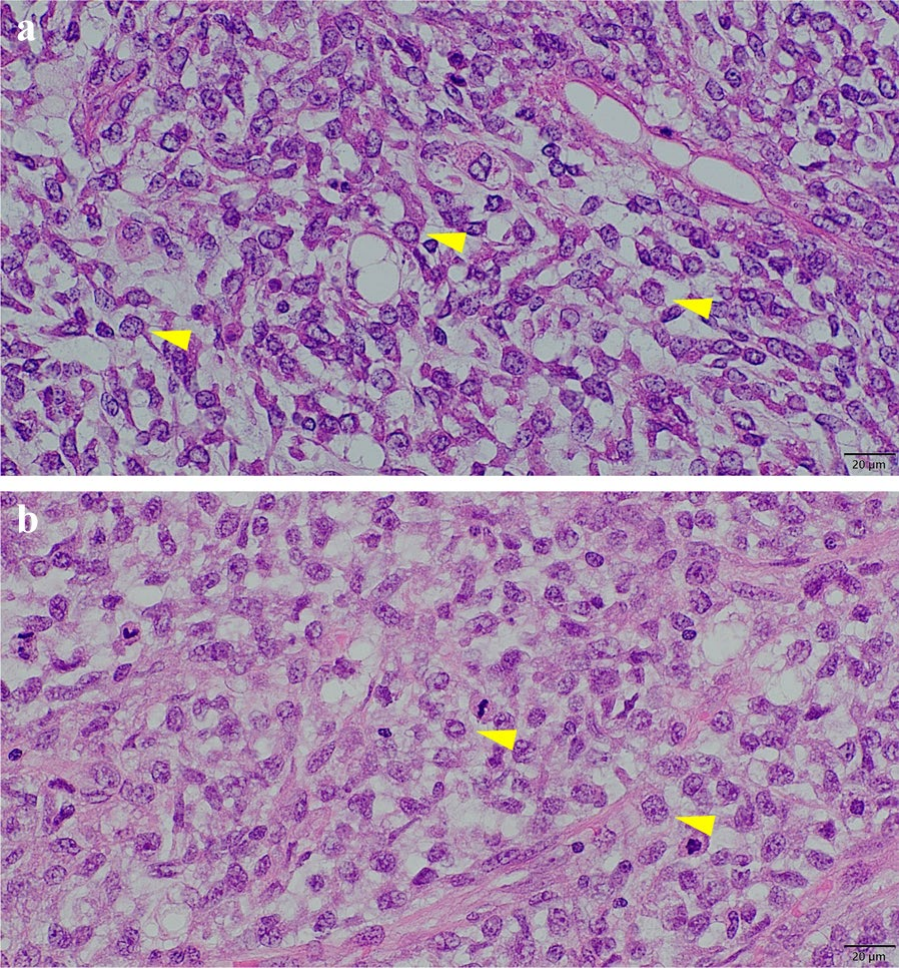
**a**, **b** Histopathological examinations showing > 5% round cells in respectively primary and metastatic tumors

The small intestine was reconstructed using a functional end-to-end anastomosis. The operative time was 118 min, and the intraoperative blood loss was approximately 10 mL. The resected specimen was a 10 × 8 × 5 cm multifocal mass (Fig. 4) with no invasion into the mucosal surface of the small intestine. Macroscopically, the tumor was located in the mesentery of the small intestine, with no invasion of the mucosa or submucosa of the small intestine. Histologically, lipoblasts containing various-sized lipid droplets were found in the tumor of the mesentery of the small intestine, the same as in the other metastases, and the milky-white tumor of the mesentery of the small intestine lacked central fatty components and was mainly composed of atypical cells with clear nucleoli and increased cell densities (Fig. 5a, b). Moreover, more than 5% round cells were found in the primary and all metastatic tumors (Fig. 6a, b). The patient was diagnosed with recurrent small intestinal mesenteric metastases from MLS. Compared with the initial surgical specimen, the same subtype was observed; however, the degree of fission was higher (3/10 HPF to 16/10 HPF), which also differed from the left cardiophrenic angle (2/10 HPF), and the right buttock (1/10 HPF). The patient had an uneventful postoperative course and was discharged on postoperative day 8. There was no evidence of distant metastases or local recurrence 12 months postoperatively.

## Discussion

There are various histological subtypes of soft tissue tumors, with benign tumors accounting for the majority. Liposarcomas are one of the most common histological types of soft tissue sarcoma (STS) [5]. According to the World Health Organization (WHO) classification, liposarcomas are categorized into five subtypes: well-differentiated, dedifferentiated, myxoid, pleomorphic, and myxoid pleomorphic [6, 7]. MLS is the second most common subtype of liposarcoma, accounting for 20––50% of liposarcomas [4]. MLS is primarily found in the extremities and rarely in the retroperitoneum. Moreover, the percentage of tissue comprising round cells is associated with a poor prognosis, and a 5% cutoff point has been reported to define high-grade MLS as a poor prognostic indicator [8].

There have been reports that more than 95% of MLS has a translocation of the FUS and DDIT3 (CHOP) genes, which is distinct from the well-differentiated and dedifferentiated forms with amplification of chromosome 12q13-15 carrying the MDM2, CDK2, and MHGA2 genes [8, 9]. The local recurrence rate of low-grade MLS after resection is low; however, unlike other STSs, MLS has metastatic potential regardless of grade. Metastases occur in 10% of cases, whereas the risk of distant disease increases to 60% in 10 years for MLS with > 5% round cells [8].

The location of MLS differs from that of other STSs. Although most STSs metastasize to the lungs, MLS more commonly metastasize to soft-tissue sites and the spine (Table 1). Additionally, the Fédération Nationale des Centres de Lutte Contre le Cancer (FNCLCC) reported a grading system for the risk of poor prognosis based on age, margin, histology, grade, and tumor size [10]. Furthermore, the number of metastases and presence of abdominal and retroperitoneal metastases are significantly associated with poor prognosis [3]. Curative resection is the most important treatment modality for MLS. Even if MLS is resected with wide clear margins, the recurrence rate is less than 10%, and a microscopically positive margin leads to a local recurrence rate of 15% [5]. Furthermore, the effectiveness of adjuvant radiotherapy or chemotherapy for MLS with round cells has been reported [5]. Although the treatments mentioned above have significantly improved the prognosis at initial diagnosis, established treatment methods have not been standardized for recurrent MLS [11].

**Table 1:** Metastatic site of MLS

	Author/Year	Site	Number of patients (%)
1	Ogose A/2000 [26]	Liver	3
		Peritoneum	4
2	Watanabe H/2001 [27]	Intraabdominal	1
3	Kenneth S/2008 [14]	Soft tissue	9 (75)
		Bone	7 (58)
		Spine	5 (42)
		Liver	4 (33)
		Lung	4 (33)
		Intraabdominal	3 (25)
		Mesentery	1 (1)
		Retroperitoneal	1 (1)
		Chest wall	1 (1)
		Pulmonary	1 (1)
4	F. Grosso/2009 [28]	Lung or pleura	17 (53)
		Abdominal cavity	14 (44)
		Soft tissue	14 (44)
		Pericardium and heart	11 (34)
		Mediastinum	10 (31)
		Bone	8 (25)
		Liver	1 (3)
5	Tomioka K/2018 [2]	Retroperitoneal	1
6	Belal S/2019 [29]	Mesentery of the small intestine	1
7	Dong-Wook K/2019 [1]	Mesentery of the small intestine	1
8	Sze Li S /2020 [4]	Greater omentum	1
9	Shinoda Y/2020 [3]	Other soft tissues	23 (47.9)
		Bone	15 (31.3)
		Abdomen or retroperitoneum	11 (22.9)
		Liver	9 (18.8)
		Lung	9 (18.8)
10	Julia D/2021 [18]	Soft tissue	26 (84)
		Pulmonary	21 (68)
		Intraabdominal	15 (48)
		Bone	14 (45)
		Lymph node	14 (45)
		Retroperitoneal	9 (29)
		Liver	6 (19)
		Heart	4 (13)
		Pancreas	4 (13)
		Brain	1 (3)
		Adrenal	1 (3)
		Colon	1 (3)
11	Our case/2023	Mesentery of the small intestine	1

Liposarcoma has a higher probability of recurrence than other STSs and differs in the site of recurrence, even in low-grade tumors [5]. The FNCLCC histologically classifies MLS grade 3 as the dedifferentiated type. The 3-year local recurrence rate of MLS is 15%, and the distant metastasis rate is 10%, in contrast to other subtypes [5]. Other indicators of poor prognosis include lung metastasis, number of liver metastases, and intra-abdominal and retroperitoneal metastases [3]. Asano proposed that tumor grade and size are associated with poor prognosis, and that lung metastases could be predictive factors for extrapulmonary metastases [12]. Histologically, round cells indicate poor prognoses [8]. In our case, we found more than 5% round cells in both the primary and metastatic tumors at recurrence, including the left cardiophrenic angle, the right buttock, and small intestinal mesentery. Although no prognostic factors have been reported for the histopathological findings of recurrent MLS, the presence of prognostic predictors for both initial and recurrent MLS could predict a high risk of recurrence.

The effectiveness of whole-spine MRI for follow-up has been described in the National Comprehensive Cancer Network (NCCN) clinical guidelines [13]. Furthermore, according to Sheah, total-body MRI, including that of the spine and bones, is useful for identifying recurrences, particularly in patients with spinal or pelvic metastases [13, 14]. Additionally, because soft tissue is typically beyond the area of surveillance, metastases are frequently discovered as recurrences, delaying diagnoses. Although Visguss reported a high rate of pulmonary metastases, secondary metastases were believed to be caused by misdiagnosis at the time of the initial recurrence [15]. According to previous reports, PET-CT is insufficient to detect recurrence [16]. In Japan, PET-CT and whole-body MRI are not recommended for surveillance. Shinoda et al. suggested that CT is appropriate twice or thrice a year for the first 4 years and then once a year for 8 years. Imaging studies, such as CT, PET-CT, and MRI for postoperative follow-up, remain controversial. Up to the first year, 80% of patients develop metastases, and by the eighth year, 90% have been detected [3]. In our case, CT and whole-spine MRI were performed for surveillance, and additional PET/CT was used to detect all metastases.

Stage I, II, and III disease-free survival rates were 86%, 72%, and 52%, respectively [17]. The overall survival rates for Stage I, II, and II disease were 90%, 81%, and 56%, respectively [17]. Metastases develop in 14–32% of patients with MLS [18]. Patient age, tumor size, tumor depth, surgical margins, and morphological factors, including grading, necrosis, mitotic rate, proliferation index, and P53 overexpression, have all been reported to affect the prognosis at the initial diagnosis of MLS [3]. According to previous reports, the most crucial factor influencing survival or the development of distant metastases is the presence of round cell components [3, 8]. In a retrospective study, Shinoda et al. reported that liver and lung metastases led to poor prognosis [3]. The NCCN guidelines recommend that metastasectomy with or without neoadjuvant or adjuvant chemotherapy and radiation can be adapted for single organs and limited tumor bulk for recurrent metastases [19]. Complete resection of lung or extrapulmonary metastases has been reported to achieve a better prognosis for multiple metastases [3]. These reports also suggest that an accurate diagnosis is crucial at the time of recurrence to prevent misdiagnosis at other metastatic sites because multiple metastases are frequently discovered, and complete resection is essential for treating recurrent liposarcoma to achieve long-term survival.

Furthermore, MLS is recognized as being more radiotherapy-sensitive than other histological subtypes of STS [3]. In addition to traditional radiation therapy using 60 Gy, radical radiation therapy uses carbon ions and photon beams [3]. According to a previous study, radiation may be an alternative to metastatic resection in some cases [3]. In contrast to other liposarcoma subtypes, MLS responds well to both chemotherapy and radiation therapy. The effectiveness of IFM as neoadjuvant chemotherapy (NAC) for MLS has been reported, and the 5-year disease-free survival has increased by 27% [20]. Unresectable recurrence has conventionally been treated with systemic chemotherapy using drugs such as doxorubicin and ifosfamide [21]. Additionally, the efficacy of pazopanib, trabectedin, and eribulin has been demonstrated [22–24]. There are few reports on the treatment of unresectable recurrent MLS. At present, complete resection, rather than chemotherapy or radiotherapy, is likely to remain important for resectable recurrent MLS.

Although recurring liposarcomas require surgical resection, relatively few cases involving laparoscopic resection have been reported [6]. MLS has a 45% recurrence rate in the setting of multiple metastases. MLS with round-cell components tends to metastasize to the retroperitoneum and lymph nodes [15]. Laparoscopy, in addition to preoperative imaging, is crucial for detecting liposarcoma metastases intraoperatively. In addition, we decided to use a laparoscopic procedure because of the small tumor size, which would result in a less invasive procedure and reduce the risk of postoperative adhesions. However, if the tumor is large or has adhesions, conversion to open surgery is necessary to prevent tumor rupture. When the location or origin of the tumor is unknown before surgery, one advantage of laparoscopy is the ability to select an appropriate incision to perform complete resection. Owing to its magnifying effect, laparoscopic surgery enables precision surgery and is helpful for total liposarcoma removal because the tumor's borders within the surrounding tissues are frequently ambiguous [25]. We reviewed four case reports of patients with recurrent MLS excised laparoscopically (Table 2). The tumors were located in the small intestine, mesentery of the small intestine, retroperitoneum, and intra-abdominal region. Metastases in all these cases were found within 12 months after the initial surgery. Although the median tumor size was 15 cm, the tumors were completely resected. The achievement of complete laparoscopic resection is likely to depend more on the location of recurrence than on the tumor size. One patient had metastases to the small intestine and liver. Our patient had metastases to three locations: the left cardiophrenic angle, right buttock, and mesentery of the small intestine. We decided to primarily resect all metastases because the locations predicted that all metastatic lesions would be resectable, despite their size. Laparoscopic surgery effectively prevented missed metastases not identified preoperatively by imaging in our patients because the primary tumor contained > 5% round cells, leading to the possibility of multiple metastases, even intra-abdominally. Laparoscopic detection may be effective for multiple metastases and recurrences in the abdomen or retroperitoneum.

**Table 2:** Cases of intra-abdominal and retroperitoneal myxoid sarcoma resected laparoscopically

Auther/Year	Sex	Age (y/o)	Imaging studies	Tumor site	Size (mm)	Surgery	Metastasis or recurrence after resection for metastatic lesion	Pathology	Other	Timeto metastases (y)
Yamashita S/2020	M	69	CT	Small intestine	36	Laparoscopic resection	None	Liposarcoma	Liver metastasis	1
Dong-Wook K/2019	M	60	CT, MRI, PET	Mesentry of the small intestine	250	Laparotomy	None	Myxoid liposarcoma	–	6
Belal S/2019	F	63	US, CT	Mesentry of the small intestine	350(gross total resection)	Laparotomy	–	Well differentiated liposarcoma	–	3
Tomioka K/2018	M	67	CT, MRI	Retroperitoneum	50	Laparoscopic resection	None	Liposarcoma	–	0.4

## Conclusion

Small intestinal mesenteric metastases from MLS are rare. Moreover, there are few reports on laparoscopic resection. In this case, the laparoscopic approach was useful for detecting the tumor location and determining the range of resection.

## Data Availability

The dataset supporting this article is available from the Department of Digestive and General Surgery, Graduate School of Medicine, University of the Ryukyus.

## Abbreviations

CT: Computed tomography
FDG: Fluorodeoxyglucose
FNCLCC: Fédération Nationale des Centres de Lutte Contre le Cancer
MLS: Myxoid liposarcoma
MRI: Magnetic resonance imaging
NCCN: National Comprehensive Cancer Network
PET: Positron emission tomography
STS: Soft tissue sarcoma
WHO: World Health Organization
